# Personal

**Published:** 1898-02

**Authors:** 


### Personal.

DR. P. W. VAN PEYMA, of Buffalo, has been appointed a member of the board of school examiners to fill the unexpired term of Dr. Conrad Diehl, elected mayor. The appointment of Dr. Van Peyma not only receives professional but general approval. He is a native of Erie county, has been a resident of Buffalo many years and is in every way qualified for the duties of the office.

DR. R. HARVEY REED, of Columbus, O., has resigned the editorship of the *Columbus Medical Journal* to accept the appointment of superintendent and surgeon-in-charge of the Wyoming General Hospital, at Rock Springs, Wyoming. Dr. Reed will retain his interest in the Columbus Medical Publishing Co., and become an associate editor of the journal.

DR. ROSWELL PARK and Mrs. Park, of Buffalo, who have been spending a number of months in Southern Europe, arrived home January 1, 1898. Dr. Park immediately resumed his professional work as well as his duties as professor of surgery at Buffalo University Medical College.

DR. F. WHITEHILL HINKEL and Mrs. Hinkel, of Buffalo, who have been in Europe for some months, returned just before Christmas last. Dr. Hinkel has resumed his practice as well as his teaching at Buffalo University Medical College, in which he is professor of clinical laryngology.

DR. W. E. DIGNEN, of Buffalo, on his return from Europe last autumn, established himself at 544 Plymouth avenue, instead of at 381 Fourteenth street, as heretofore mentioned.

